# Perturbations in the Carotenoid Biosynthesis Pathway in Tomato Fruit Reactivate the Leaf-Specific Phytoene Synthase 2

**DOI:** 10.3389/fpls.2022.844748

**Published:** 2022-02-25

**Authors:** Uri Karniel, Nastacia Adler Berke, Varda Mann, Joseph Hirschberg

**Affiliations:** Department of Genetics, Alexander Silberman Institute of Life Sciences, The Hebrew University of Jerusalem, Jerusalem, Israel

**Keywords:** carotenoid biosynthesis, phytoene synthase, *Solanum lycopersicum*, fruit, chromoplast, retrotransposons

## Abstract

The accumulation of the red carotenoid pigment lycopene in tomato (*Solanum lycopersicum*) fruit is achieved by increased carotenoid synthesis during ripening. The first committed step that determines the flux in the carotenoid pathway is the synthesis of phytoene catalyzed by phytoene synthase (PSY). Tomato has three *PSY* genes that are differentially expressed. *PSY1* is exclusively expressed in fruits, while *PSY2* mostly functions in green tissues. It has been established that PSY1 is mostly responsible for phytoene synthesis in fruits. Although PSY2 is found in the chromoplasts, it is inactive because loss-of-function mutations in *PSY1* in the locus *yellow flesh* (*r*) eliminate carotenoid biosynthesis in the fruit. Here we demonstrate that specific perturbations of carotenoid biosynthesis downstream to phytoene prior and during the transition from chloroplast to chromoplast cause the recovery of phytoene synthesis in *yellow flesh* (*r*) fruits without significant transcriptional changes of *PSY1* and *PSY2*. The recovery of carotenoid biosynthesis was abolished when the expression of *PSY2* was silenced, indicating that the perturbations of carotenoid biosynthesis reactivated the chloroplast-specific PSY2 in fruit chromoplasts. Furthermore, it is demonstrated that PSY2 can function in fruit chromoplasts under certain conditions, possibly due to alterations in the plastidial sub-organelle organization that affect its association with the carotenoid biosynthesis metabolon. This finding provides a plausible molecular explanation to the epistasis of the mutation *tangerine* in the gene *carotenoid isomerase* over *yellow flesh*.

## Introduction

**Figure 1:**
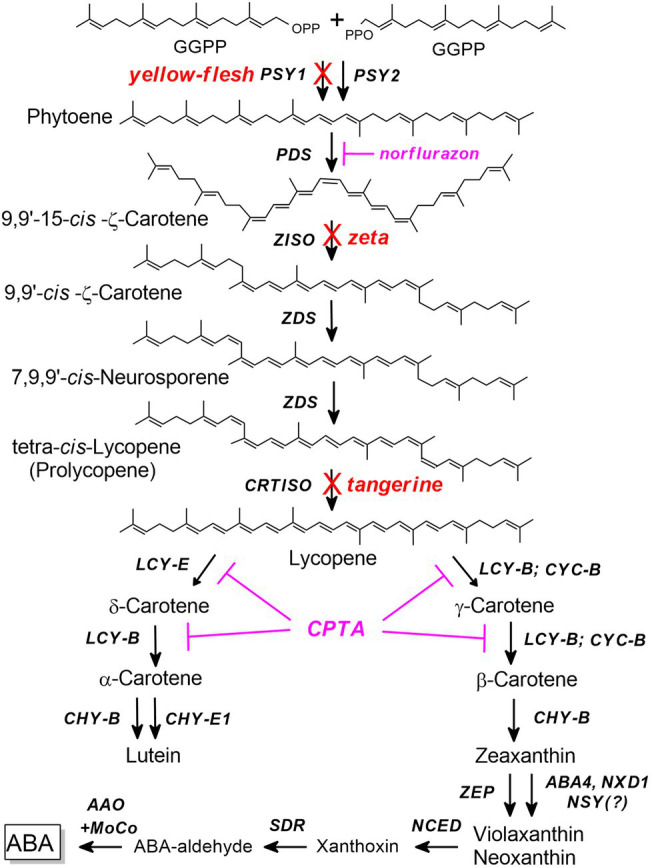
The carotenoid biosynthesis pathway in tomato. AAO, abscisic aldehyde oxidase; ABA, abscisic acid; ABA4, involved in neoxanthin synthesis; CHY-B, β-carotene hydroxylase, CHY-E- ε-carotene hydroxylase, CRTISO, carotene isomerase; CYC-B, chromoplasts-specific lycopene β-cyclase; GGPP, geranylgeranyl diphosphate; LCY-B, lycopene β-cyclase; LCY-E, lycopene ε-cyclase; MoCo, molybdenum cofactor; NCED, 9-cis-epoxycarotenoid dioxygenase; NSY, presumed neoxanthin synthase; NXD1, involved in neoxanthin synthesis; PDS, phytoene desaturase; PSY, phytoene synthase; SDR, short-chain alcohol dehydrogenase/reductase; ZDS, ζ-carotene desaturase; ZEP, zeaxanthin epoxidase, ZISO, ζ-carotene isomerase. Mutations that interrupt the pathway are indicated in red and inhibitors in magenta.

Carotenoid pigments are naturally occurring isoprenoid molecules that perform essential functions in plants and animals (Cuttriss et al., 2011). They are indispensable in all photosynthetic organisms where they play roles in photosynthesis, both in light-harvesting and protection against damages caused by excessive light energy (Telfer, 2005; Hashimoto et al., 2016). Carotenoids are vital components in human nutrition for their health benefits as antioxidants and precursors of vitamin A (Eggersdorfer and Wyss, 2018; Rodriguez-Concepcion et al., 2018; Von Lintig and Quadro, 2020). In addition to furnishing flowers and fruits with colors, carotenoids also serve as precursors in the biosynthesis of growth regulators and developmental signals, such as abscisic acid (ABA) and strigolactone (Walter et al., 2015; Hou et al., 2016; Wang et al., 2020). In plants, carotenoids are synthesized in plastids from isopentenyl pyrophosphate (IPP)-derived geranylgeranyl diphosphate (reviewed in: Hirschberg, 2001; Ruiz-Sola and Rodríguez-Concepción, 2012; Nisar et al., 2015; Rosas-Saavedra and Stange, 2016). The first committed step in the biosynthesis pathway is phytoene formation from two molecules of geranylgeranyl pyrophosphate (GGPP) catalyzed by the enzyme PSY (Figure 1). In the subsequent steps, the production of all-*trans*-lycopene from 15-*cis*-phytoene involves four enzymes: phytoene desaturase (PDS), ζ-carotene isomerase (ZISO), ζ-carotene desaturase (ZDS), and carotenoid isomerase (CRTISO; Figure 1). PDS and ZDS introduce four double bonds by catalyzing two symmetric dehydrogenation reactions to yield two *cis*-configured molecules, ζ-carotene and lycopene, respectively (Isaacson et al., 2004). ZISO is required for *cis*-to-*trans* conversion of the 15–15′ *cis* double bond in tri-*cis*-ζ-carotene (Chen et al., 2010). Another isomerase, CRTISO, produces all-*trans*-lycopene from tetra-*cis*-lycopene (“prolycopene”; Isaacson et al., 2002; Park et al., 2002). Next, the linear molecule lycopene undergoes cyclization either by LCY-b or CYC-b to create a β-ring or LCY-e to form an ε-ring (Cunningham et al., 1996; Pecker et al., 1996). Hydroxylation of the cyclized carotenes produces the xanthophylls (oxygenated carotenoids) lutein in the ε-branch, and zeaxanthin, violaxanthin, and neoxanthin in the β-branch.

The tomato (*Solanum lycopersicum*) is a leading model in fleshy fruit ripening research and a favorite plant for studying the regulation of carotenoid biosynthesis (reviewed in: Fray and Grierson, 1993b; Fraser et al., 1994; Giovannoni, 2001; Bramley, 2002; Alexander and Grierson, 2002; Giovannoni, 2007; Klee and Giovannoni, 2011; Liu et al., 2015; Li et al., 2019). During the first 5–7 weeks after anthesis, the developing tomato fruit is green as its tissues contain chloroplasts. At the “breaker” stage of ripening, the mature green fruit undergoes a dramatic color change due to the degradation of chlorophyll and a concomitants accumulation of lycopene. Increased synthesis and accumulation of carotenoids in high concentration are linked to a chloroplast-to-chromoplast transition (Maass et al., 2009; Lado et al., 2016; Sun et al., 2018; Llorente et al., 2020), and involves extensive changes in gene expression (Alba et al., 2005; Klee and Giovannoni, 2011; Giovannoni et al., 2017). The synthesis of phytoene is a rate-limiting step that determines the flux of carotenoid biosynthesis. Therefore, PSY is a key regulating enzyme of the pathway (Bramley, 2002; Rodriguez-Villalon et al., 2009; Ruiz-Sola and Rodríguez-Concepción, 2012; Enfissi et al., 2017). While *Arabidopsis thaliana* has a single *PSY* gene, in some other plant species *PSY* constitutes a small gene family that are distinctly expressed in different organs (Gallagher et al., 2004; Giorio et al., 2008; Li et al., 2008; Welsch et al., 2008; Li et al., 2009; Arango et al., 2010; Qin et al., 2011; Lopez-Emparan et al., 2014; Ahrazem et al., 2019; Wei et al., 2020; Wang et al., 2021). The tomato genome contains three *PSY* genes that show distinct expression patterns (Fraser et al., 2007; Giorio et al., 2008). The amino acid sequences of PSY1 and PSY2 are highly conserved. However, although they have similar enzymatic properties, they differ in cofactor requirement and K_m_ for GGPP (Fraser et al., 2000). It was demonstrated that PSY2 is enzymatically more efficient than PSY1 when expressed in Arabidopsis leaves or *E. coli* (Cao et al., 2019). *PSY2* functions in leaves and other green tissues that contain chloroplasts (Giorio et al., 2008). *PSY3* is expressed in roots where the carotenoid-derived hormones abscisic acid (ABA) and strigolactones are synthesized (Walter et al., 2015; Stauder et al., 2018). The gene responsible for carotenoid biosynthesis in chromoplast-containing fruits is *PSY1*. Its transcription is upregulated at the “breaker” stage of fruit ripening and remains high till the ripe stage. Although *PSY2* is also expressed to some extent in the fruit, it does not contribute to carotenoid synthesis in this organ (Fraser et al., 1999). This is evident in the recessive mutation *yellow flesh* that impairs the *PSY1* gene and abolishes phytoene synthesis in fruits (Fray and Grierson, 1993a; Fraser et al., 1999; Kachanovsky et al., 2012). Fruits of the *yellow flesh* mutant are yellow due to the pigment naringenin chalcone, and they contain negligible amounts of carotenoids. Therefore, the locus was named *r* (R for red, r for non-red or yellow; McCue, 1952). The oldest known *yellow-flesh* allele, named *r*^2997^, was described as a “spontaneous” mutation (Chetelat, 2002). It has been shown that this allele eliminates the transcription of *PSY1* in fruit (Kachanovsky et al., 2012). Another mutation, *tangerine* (locus *t*), has orange fruit flesh due to the accumulation of tetra-cis-lycopene (“prolycopene”) as a result of a mutation in the gene encoding the CRTISO enzyme (Isaacson et al., 2002). Because this reaction occurs downstream to phytoene in the carotenoid biosynthesis pathway (Figure 1), blocking phytoene synthesis by *yellow flesh* should be epistatic to *tangerine*. However, several studies in the 1950s reported that the mutation *tangerine* is epistatic to *yellow-flesh r*^2997^ since the phenotype of the double mutant *tr* was typical of *tangerine* (Tomes et al., 1953). Previously, we have demonstrated that under the genetic background of *tangerine*, transcription of *PSY1* in *r*^2997^ was partially recovered (Kachanovsky et al., 2012). It was suggested that this phenomenon was induced by the increased concentration of *cis*-carotenes (Kachanovsky et al., 2012). However, the molecular mechanism underlying the epistasis of *tangerine* over *r*^2997^ has remained unclear because the exact nature of the mutation in this allele has not been identified. Here we characterize the mutation in *yellow flesh r*^2997^ caused by an insertion of a retrotransposon in the coding region of *PSY1* and describe its effects on the transcription of the gene. Characterization of additional *yellow flesh* alleles and obstructing the carotenoid biosynthesis pathway downstream to phytoene indicate that the epistasis phenomenon is mainly due to the activation of the chloroplast phytoene synthase, PSY2, otherwise inactive in tomato fruit.

## Materials and Methods

### Plant Material and Growth Conditions

The tomato (*Solanum lycopersicum*) varieties M82 and Rutgers served as a reference “wild-type.” The *tangerine t*^3002^ and *yellow flesh r*^2997^ mutants and the wild species *S. pimpinellifolium* accession LA1589 were obtained from the Tomato Genetics Resource Center (University of California, Davis, CA). Mutants *t*^3406^, *r*^3756^, and *z*^2803^ were isolated from the variety M82 following mutagenesis and screening (Menda et al., 2004). Introgression line IL3-2, which carries a single chromosomal segment from *S. pennellii* (LA716) in the genetic background M82 (Eshed and Zamir, 1995), was obtained from Prof. Zamir (The Hebrew University of Jerusalem, Israel). Virus-induced gene silencing (VIGS) experiments were carried out in a transgenic Moneymaker line overexpressing the *Delila* (*Del*) and *Rosea1* (*Ros1*) Myb transcription factors from *Antirrhinum majus* under control of the fruit-specific promoter E8 (Butelli et al., 2008; Orzaez et al., 2009). Plants were grown in the greenhouse as previously described (Neuman et al., 2014).

### DNA Extraction and Genotyping

DNA was extracted from approximately 15 mg of young leaves as previously described (Eshed and Zamir, 1995). Homozygous F2 plants from crosses between the different carotenoid mutants were identified by visual screening and confirmed through DNA genotyping. The *tangerine* alleles *t*^3406^ and *t*^3002^, and the *zeta* mutant *z*^2083^ were identified by the virescence appearance of their shoots and the tawny flower color typical of *tangerine*. The genotyping of the homozygous *yellow flesh* mutants IL3-2 (*r*^sp^), *r*^2997^, *r*^3756^, and *Delila + Rosea* (DR) was confirmed by polymerase chain reactions (PCR) amplification using the following primers: For *PSY1* in IL3-2, 5′-AATACTTTTAGGGTCAAACAATTAA-3′ (forward) and 5′-AAAAATTGACCCACATTGAAAAA-3′ (reverse). Due to a deletion of 672 bp in the *PSY1* from *S. pennellii* and its introgression line IL3-2, the PCR amplification yielded a 1065 bp fragment in the wild-type tomato *S. lycopersicum* and 393 bp in the IL3-2 introgression line. For *PSY1* in *r*^2997^, the primers 5′-CGGGAGTCATTAGCATAGTTCC-3′ (forward) and 5′- CGAGGCATAGGAATTTGGTG-3′ (reverse) were used for PCR amplification. A 5 kb insertion in the *PSY1* from *r*^2997^ generates a fragment of 5,326 bp in *r*^2997^ and 447 bp in the wild-type. Amplification of such a large fragment by PCR was possible using the KAPA HiFi HotStart ReadyMix kit #KK2601 (Roche). For *PSY1* in *r*^3756^, 5′-CGAGGCATAGGAATTTGGTG-3′ (forward) and 5′-ACCTATCTAAGGCTGCCGGGGTAATA-3′ (reverse). The PCR product was sequenced to identify the presence of a transition mutation changing codon 151 from Trp to an early stop codon (Kachanovsky et al., 2012).

The presence of the transgenic *DR* genes was confirmed in seedlings using PCR amplification with the following primers: 5′AAGGCTTCTGATACGGACAAG3′ (forward) and 5′TCTTACGGCTTCCATCACTTC3′ (reverse).

### RNA Extraction and Quantification

Total RNA was extracted from 200 mg fruit tissue with TRI Reagent RNA isolation reagent (Sigma-Aldrich), according to the manufacturer’s protocol. For cDNA preparation, reverse transcription was done with the iScript™ gDNA Clear cDNA Synthesis Kit #172–5,035 (Bio-Rad). Rapid amplification of 5′ cDNA end (5′-RACE) analyses to determine the 5′ end of the transcripts of *PSY1* were carried out with SMART^®^ RACE 5′/3′ Kit from Clontech Laboratories Inc. (Mountain view, Ca, United States) according to the manufacturer’s protocol. The reverse primer was 5′-TCCATACGCATTCCTTCAATC-3′ (exon #7).

To measure transcript levels of *PSY1* and *PSY2*, the cDNA was amplified using ProFlex PCR System (Applied Biosystems by Thermo Fisher Scientific) in a quantitative PCR protocol using the Applied Biosystems^™^ Fast SYBR^™^ Green Master Mix on a StepOnePlus^™^ Real-Time PCR System (Applied Biosystems). Cycling conditions were 95°C for 20 s, followed by 40 cycles of 95°C for 3 s, 60°C for 30 s and fluorescence acquisition at 60°C. For each gene, the relative mRNA level was determined in three biological replicates. The primers used for the RT-PCR amplifications were 5′-AACTTGTTGATGGCCCAAAC-3′ (forward) and 5′-CTGTATCGGACAAAGCACCA-3′ (reverse), for *PSY1* (Solyc03g031860); 5′-AGTTCTGCTAGTAGATGGCC-3′ (forward) and 5′-GGGCACTAGAGATCTTGCAT-3′ (reverse) for *PSY2* (Solyc02g081330); and 5′-AACAGTTGGCCTAATGAGTGTGC-3′ for *PSY3* (Solyc01g005940) The ACTIN gene (Solyc11g005330) served as a control for normalization, using the primers 5′-TTGCTGACCGTATGAGCAAG-3′ (forward) and 5′-GGACAATGGATGGACCAGAC-3′ (reverse) that differentiate between genomic DNA and cDNA sequences.

### Functional Assay of PSY1 Transcripts

To investigate the enzymatic activity of the different PSY1 variants from *r*^2997^, the complete cDNA of this gene was obtained from RNA isolated from pulp of fresh fruit of *r*^2997^ followed by RT-PCR using the primers 5′-AGCC**ACTAGT**TGGCTTGTTGAGTGAAGCATAT-3′ (forward) and 5′-GTCG**CTCGAG**CTCATGCTTTATCTTTGAAGAGAGG-3′ (reverse). The PCR products were cloned into the plasmid pBluescript SK^+^ between the SpeI and XhoI restriction sites, and the plasmids were transfected into *Escherichia coli* cells of the strain XL1Blue. Sequencing of different bacterial colonies obtained from this cloning revealed two variants that can produce possible functional open reading frames of *PSY1*. One of these variants resulted from skipping over exon #4 of PSY1 while the other from skipping over exon #2 and exon #4 of this gene. Thus, these two new plasmids were termed pPSY1V1 and pPSY1V2, respectively. As a control, the full-length transcript was amplified from wild-type tomato fruit and cloned into the plasmid pBluescript SK^+^ between the SpeI and XhoI sites, generating the pfull_PSY1 vector. The inserts were sequenced to identify possible PCR-derived mutations.

The plasmid pACCRT-EIB carries the *Pantoea agglomerans* genes Idi, crtE, crtI, and crtB that produce lycopene when expressed in *E. coli* (Cunningham and Gantt 2007). The *crtB* gene, which encodes phytoene synthase, was knocked out pACCRT-EIB through site-directed mutagenesis using the protocol of QuikChange^®^ Site-Directed Mutagenesis Kit of Stratagene (La Jolla, CA) with the KAPA HiFi HotStart ReadyMix enzyme with the primers 5′-TCAGGAAGTGGCTATGCTCATGATATCGCCCC-3′ (forward) and 5′-GGGGCGATATCATGAGCATAGCCACTTCCTGA-3′ (reverse). The new plasmid was called pACCRT-EI. Plasmids pPSY1V1, pPSY1V2, and the full-length *PSY1* cDNA plasmid pfull_PSY1, were co-transfected with pACCRT-EI to *E. coli* strain XL1-Blue grown on Luria-Bertani (LB) medium containing the antibiotics ampicillin and chloramphenicol. To enhance the expression of these genes, 24 mg/l of Isopropyl 1-thio-β-D-galactopyranoside (IPTG) was added to the LB medium. *E. coli* cells were grown overnight at 37°C on LB agar plates followed by 5 days at room temperature for pigment accumulation.

### Pigment Extraction and Analysis

Fresh samples of fruit were collected from three biological replicates. Fruit pigments were extracted from 200 to 250 mg of fresh pericarp tissue at the “breaker” and “ripe” (breaker plus 7 days) stages. The tissue was ground in 1 ml of 1:1 water chloroform mixture. The chloroform phase was separated by centrifugation and dried under a stream of N_2_. The dried carotenoid extracts were dissolved in 300 μl acetone. Carotenoids were separated by high-performance liquid chromatography analysis as previously described (Karniel et al., 2020).

#### Chemical Inhibition of Phytoene Desaturase and Lycopene Cyclase

Inhibition of phytoene desaturase (PDS) was achieved with the inhibitor norflurazon (4-chloro-5-(methylamino)-2-[3-(trifluoromethyl)phenyl]pyridazin-3-one), or its commercial formulation herbicide Zorial (Sandmann et al., 1989). For inhibition in fruits, a volume of 500–700 μl of 100 μM Zorial was injected into mature green fruits and analyzed at the ripe stage. Inhibition of PDS in fruit pericarp *ex-planta* was carried out with 100 μl of norflurazon/Zorial externally laid on pericarp disks taken from fruits at the mature green stage, as previously described (Pankratov et al., 2016). Inhibition of the enzymes LCY-B and CYC-B was performed with the inhibitor 2-(4-Chlorophenylthio)-triethylamine hydrochloride (CPTA). One milliliter of CPTA at a concentration of 100 μM was injected into mature green fruits with a 0.4 mm X 20 mm needle. Fruits with yellow and red sectors were observed at the ripe stage 4–7 days following the injection. The different pericarp sectors were dissected for carotenoid and transcript measurements.

### Silencing of the Gene PSY2

A VIGS experiment was established as previously described (Fantini et al., 2013). The pTRV2_DR plasmid was kindly supplied by Dr. Giovanni Giuliano, ENEA, Italy. A *PSY2*-specific silencing sequence was amplified with the primers 5′-**GGGGACAAGTTTGT ACAAAAAAGCAGGCT**GACGTTGCCCATTGCTTATGC-3′ (forward) and 5′-**GGGGACCACTTTGTACAAGAAAGCTGGGT**ACTCAAATGAAGTCAATTATC-3′ and cloned in the pTRV2_DR using the Gateway BP Clonase II enzyme mix and Gateway LR Clonase II enzyme according to the manufacturer’s protocol.

#### Statistical Analysis

Statistical analysis of the parameters measured in field trials was performed using JMP 15 software (SAS Institute). Mean values of the parameters of the different tested genotypes were compared using the “Fit Y by X” function and “Compare all pairs” (Tukey–Kramer).

## Results

### Molecular Characterization of the Mutation *Yellow-flesh r*^2997^

Previous studies suggested that the locus *yellow flesh r*^2997^ is genetically linked to the *PSY1* gene in chromosome #3. However, the molecular basis of the mutation has remained unknown (Kachanovsky et al., 2012). To find the *r*^2997^ mutation, a mapping population of 310 F2 plants was created from a cross between the homozygous *r*^2997^ tomato line LA2997 in the Rutgers variety genetic background and the red-fruited wild species *S. pimpinellifolium*. The mapping relied initially on two polymorphic markers between *S. pimpinellifolium* and *S. lycopersicum*, named INDEL1 and INDEL2, found in the genomic sequence spanning ~1600 kb around the *PSY1* gene in chromosome #3 (Supplementary Table S1; Supplementary Figure S1). Sixty recombinant plants between these markers were phenotyped for fruit color and were further screened with additional fourteen PCR markers (Supplementary Table S1). Due to the recessive nature of the mutation, heterozygous red-fruited plants were self-pollinated, and fruit color was checked in the F3 generation (Supplementary Figure S1A). The association between fruit phenotypes and the DNA marker results enabled us to localize the *r*^2997^ mutation within 24 kb between markers PSY1-3′ and 8,587 that spanned the whole *PSY1* gene (Supplementary Figure S1B). Using PCR amplification with primers located in this region, we have amplified an insertion of 4,867 bp in exon #4 of the *PSY1* gene in *yellow flesh* allele *r*^2997^. The nucleotide sequence of the insertion (Supplementary Figure S2) is identical to a known Ty1*-copia*-like retrotransposon of the *Rider* family (GenBank: EU195798.2; Cheng et al., 2009), which contains 397 nucleotide long terminal direct repeats (LTR) at both borders, an open reading frame encoding a Ty1-*copia*-type polyprotein, and a primer-binding site (PBS) and polypurine tract site (PPT). Interestingly, the 5′ end of the retrotransposon sequence is identical to the previously published sequence of GTOM5, an abortive mRNA transcript of *PSY1* from the mutant *yellow flesh r* in the tomato variety Ailsa Craig (Fray and Grierson, 1993a).

### Effects of the Retrotransposon on the Expression of *PSY1*

**Figure 2:**
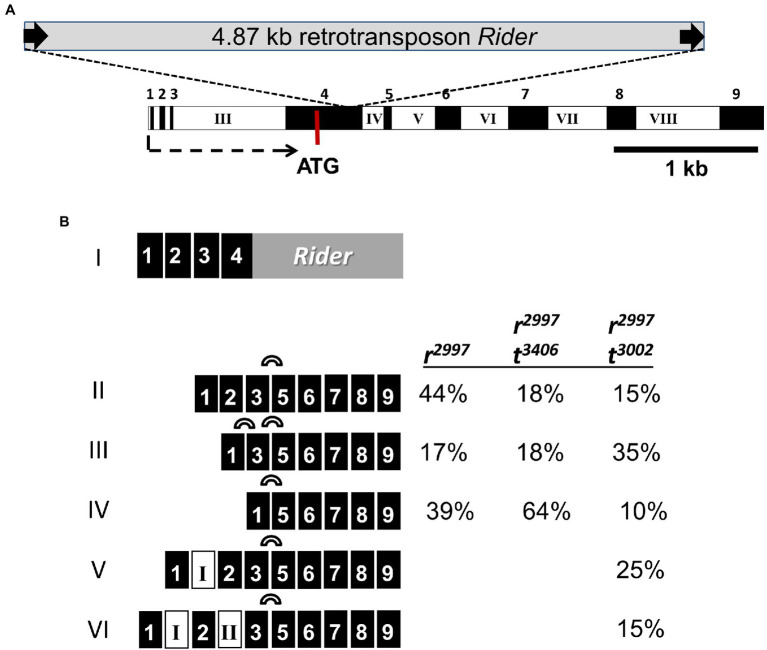
The *PSY1* gene in the mutant *r*^2997^ and its transcripts. **(A)** Genomic structure of *PSY1* with nine exons (filled boxes) and eight introns. The *Rider* retrotransposon is located in exon #4. Arrow indicates transcription initiation. **(B)** *PSY1* transcript variants detected in fruits of mutant *r*^2997^ and double mutants *r*^2997^/*tangerine*. The proportions of the various transcripts were calculated from the total transcripts that contained exons 5–9.

A rapid amplification of 5′ cDNA ends (5′-RACE) analysis was carried out to determine the transcription initiation site of *PSY1* in the wild-type tomato variety. The results showed that 81 percent of the transcripts started in the first exon and the others in exon #4 (Supplementary Figure S3). In the fruits of mutant *r*^2997^, all the *PSY1* transcripts were initiated in exon #1 (Figure 2). The primary one was a fusion of exon #4 with the retrotransposon sequence (transcript I in Figure 2). This fused transcript creates an early stop codon, similar to the one described in GTOM5 from *yellow flesh r* in the Ailsa Craig variety (Fray and Grierson, 1993a). Therefore, we conclude that *r*^2997^ in LA2997 and *r* in Ailsa Craig are essentially the same *yellow flesh* allele derived from the ancestral yellow-fruited tomato line. In addition to the most abundant abortive *PSY1* transcript, fruits of *r*^2997^ produce additional transcripts resulting from alternative splicing events (Figure 2). These transcript variants lack the retrotransposon-containing exon #4 and include exons 5–9, which encode the C-terminus of the PSY1 protein where the catalytic domain is located (Cao et al., 2019). The functionality of the alternatively spliced variants was tested in *E. coli* cells. For this purpose, the cDNA of variants II and III (Figure 2), which contain the coding sequence of exons 5–9, and the normal cDNA sequence of exons 4–9 were cloned in the plasmid pBluescript SK^+^. These plasmids were transfected into *E. coli* cells carrying plasmid pACCRT-EI, which encodes the bacterial genes CrtE and CrtI for geranylgeranyl diphosphate synthase and phytoene desaturase, respectively. In the presence of phytoene, these enzymes can synthesize lycopene. As expected, the *E. coli* cells that expressed the full-length PSY1 were red due to lycopene accumulation. In contrast, PSY1 variants lacking exon #4 are not translated into enzymatically active proteins, as the alternatively spliced PSY1 variants II and III gave rise to colorless bacteria that did not produce carotenoids (Supplementary Figure S4). These results show that PSY1 variants lacking exon #4 are enzymatically inactive, as is evident in fruits of the mutant *yellow flesh r*^2997^.

### Epistasis in the Carotenoid Biosynthesis Pathway in Tomato Fruit

The epistasis phenomenon found in the double mutant *yellow flesh r*^2997^/*tangerine* (Kachanovsky et al., 2012) was further investigated in various allele combinations. Fruits of the introgression line IL3-2, which carries the *PSY1* gene from *S. pennellii* in the variety M82, are yellow and lack carotenoids due to a substantial reduction in *PSY1* expression compared to its isogenic cultivated line M82 (Liu et al., 2003; Joung et al., 2009). Therefore, the *PSY1* gene from *S. pennellii* in the genetic background of the cultivated tomato can be considered as a *yellow flesh* allele, *r*^sp^. The IL3-2 line was crossed with the isogenic *tangerine t*^3406^ and *zeta z*^2803^ mutants, which are impaired in the genes *CRTISO* and *ZISO*, respectively, to produce in F2 generation double mutants IL3-2 *r*^sp^/*t*^3406^ and IL3-2 *r*^sp^/*z*^2803^. The genotypes of these mutants were confirmed with DNA markers. In agreement with previous observations of the *yellow flesh* allele *r*^2997^, fruit of the double mutant IL3-2 *r*^sp^/*t*^3406^ accumulated carotenes, though at a lower level than the wild type, mainly in *cis*-configurations (Table 1). These results confirm that the epistasis of *tangerine* over *yellow flesh* is a general phenomenon and is not confined to a specific *r* allele. Another *yellow flesh* allele, *r*^3756^, which was isolated by mutagenesis in the tomato variety M82, contains an early stop codon in exon #4 of the *PSY1* gene (Kachanovsky et al., 2012). Fruits of the double mutant *r*^3756^_/_*t*^3002^ were orange due to the accumulation of low levels of carotenes (Table 1 and Supplementary Figure S5). These results indicate that *tangerine* is also epistatic over the null *yellow flesh* allele *r*^3756^. As previously reported (Kachanovsky et al., 2012), the epistasis phenomenon is linked to the CRTISO impairment but not to the ZISO function loss because fruits of the double mutant IL3-2 *r*^sp^/*z*^2803^ were yellow and contained only trace amounts of carotenoids (Table 1).

**Table 1:** Carotenoid composition in ripe fruit of various tomato lines (μg g^−1^ FW).

	Lutein	Lycopene	Prolycopene	Neurosporene	ζ-Carotene	β-Carotene	Phytoene + Phytofluene	Total
*r* ^2997^	0.5 ± 0.1	0.1 ± 0.1		0.2		0.5 ± 0.1		1.3 ± 0.1
*t* ^3406^	0.5		20.2 ± 5.5	8.3 ± 1.3	17.6 ± 7.7		38.5 ± 10.2	85.6 ± 15.3
*Z* ^2803^	1.9 ± 0.2	5.8 ± 1.9			37.9 ± 6.2		59 ± 10.1	104.7
*r*^2997^/*t*^3406^		0.5	8.9 ± 2.6	1.7 ± 0.7	1.8 ± 1		3.1 ± 0.9	16 ± 5.4
IL3-2 (*r^sp^*)	1.5 ± 0.2	0.4 ± 0.3				0.9		2.8 ± 0.1
IL3-2(*r^sp^*)/*t*^3406^	0.7 ± 0.1	1.1 ± 0.3	5.2 ± 2.6	2.3 ± 0.1	6.5 ± 2.5	1.0 ± 0.1	10.4 ± 4.4	27.2 ± 10.2
IL3-2(*r^sp^*)/*z*^2803^	1.2 ± 0.3	0.4 ± 0.4		0.2 ± 0.1		0.4 ± 0.2		2.2 ± 0.5
*r* ^3756^	0.6 ± 0.1			0.3 ± 0.1		0.2 ± 0.1		1.7 ± 0.4
*r* ^3756^_/_ *t* ^3002^	0.3	0.4 ± 0.4	4.1 ± 0.9	1.5 ± 0.5	1.5 ± 0.5	0.1 ± 0.1	1.4 ± 0.5	9.4 ± 2.6
M82	0.4 ± 0.2	45.8 ± 7.3				1 ± 0.2	8.8 ± 2.8	56.9 ± 10.1

**r*^2997^ and *r*^3756^ are yellow flesh alleles; *t*^3406^ and *t*^3002^ are tangerine alleles; *z*^2803^ is a null allele of zeta. M82 represents the wild type*.

**Figure 3:**
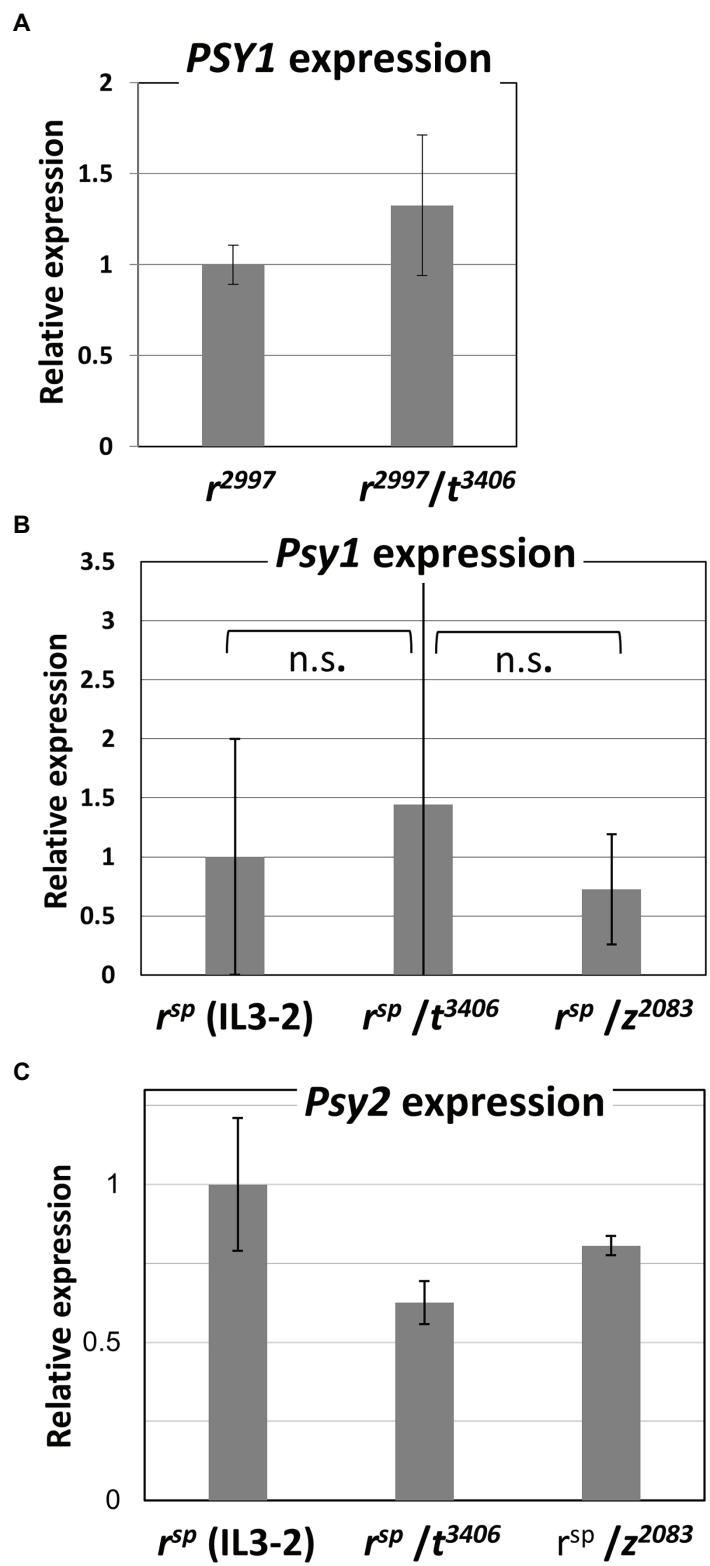
Expression of *PSY* genes in fruits. **(A)** *PSY1* transcript levels at the “breaker” stage of *r*^2997^ and *r*^2997^/*t*^3406^; **(B)** *PSY1* transcript levels at the “breaker” stage of *r^sp^*, *r^sp^*/*t*^3406^ and *r^sp^* (introgression line IL3-2)/*z*^*s*2083^; **(C)** *PSY2* transcript levels in *r^sp^*, *r^sp^*/*t*^3406^ and *r^sp^*/*z*^2083^. The data were compared using the “Compare all pairs” (Tukey–Kramer), *p* < 0.05. n.s. no significance.

Previous results have shown that the mutation *tangerine* leads to a substantial increase in the transcription of *PSY1* in *r*^2997^ fruit compared to the single mutant *r*^2997^ (Kachanovsky et al., 2012). To analyze the effect of *tangerine* on the expression of *PSY1*, we established several new qRT-PCR protocols with primers for exon #6 to measure the mRNA level of *PSY1*. The results confirmed that the transcript levels of *PSY1* in the double mutant *tangerine*/*r*^2997^ appeared to be higher than in *r*^2997^, however, to a lower extent than previously reported (Figure 3A). The increase of *PSY1* transcript in IL3-2(*r^sp^*)/*t*^3406^ compared with IL3-2(*r^sp^*) was relatively modest, but the data were not significant in this case (Figure 3B). Expression of *PSY2* in fruits of the *yellow flesh*/*tangerine* double mutants was lower than in the *yellow flesh* alleles *r*^2997^, *r*^3756^, and IL3-2 (*r^sp^*; Figure 3C). In compliance with the *PSY3* expression pattern in tomato,^1^ the root-specific *SlPSY3* (Solyc01g005940) could not be detected with qRT-PCR in fruits of any of these lines.

### Activation of Carotenoid Biosynthesis in *Yellow Flesh* Fruits by Inhibition of Carotenoid Biosynthesis Downstream of Phytoene

**Figure 4:**
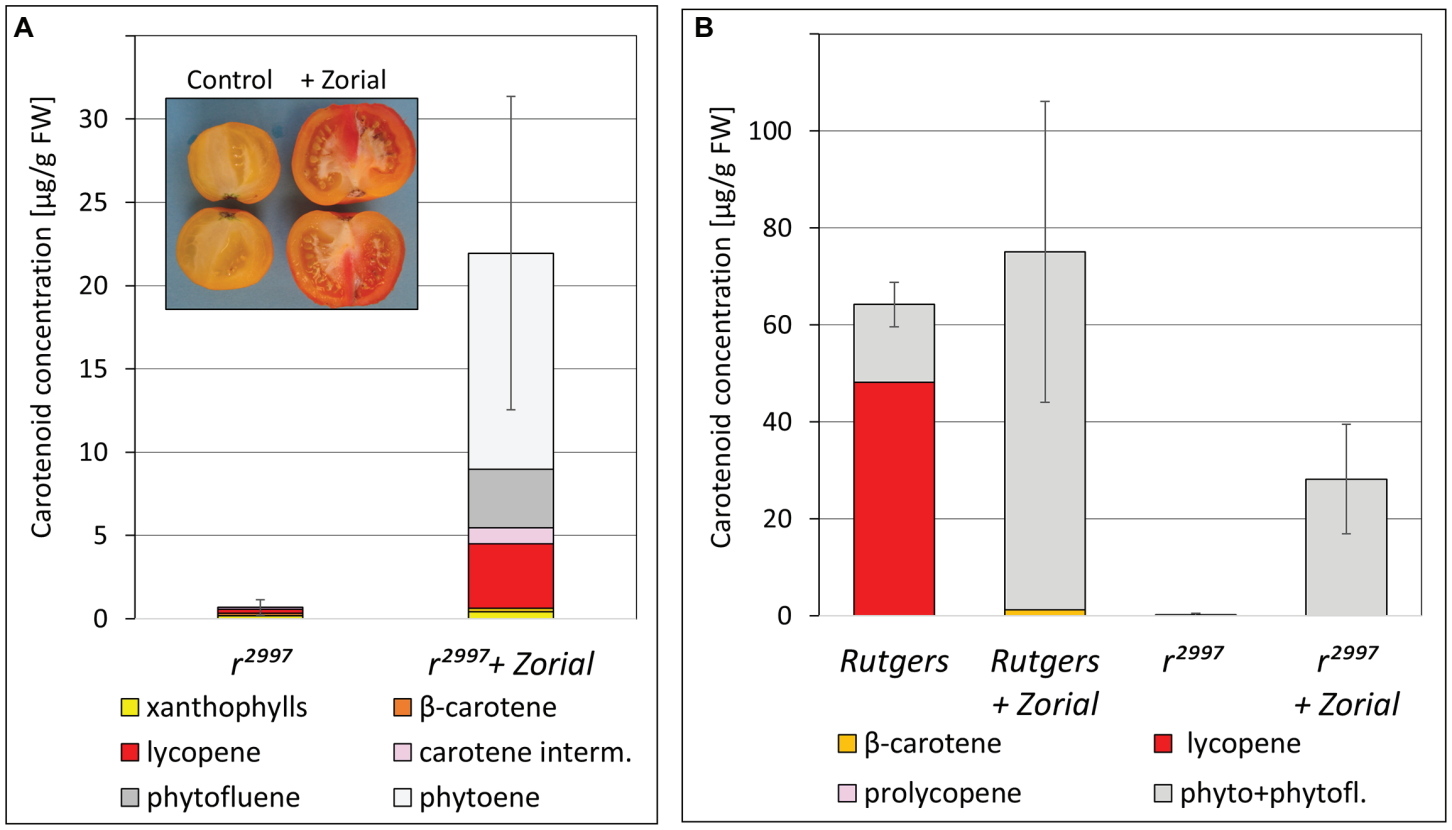
Effects of norflurazon (Zorial) on pigment accumulation in the fruit of *yellow flesh r*^2997^ (Rutgers variety background). **(A)** Carotenoid composition in fruits after Zorial injection. **(B)** Carotenoid composition in pericarp explants from Rutgers (wild type) and mutant *r*^2997^ treated with Zorial (Z).

Since *tangerine* obstructs carotenoid biosynthesis at the isomerization of *cis*-lycopene, we investigated the effects on phytoene synthesis of other pathway inhibitors. Norflurazon (Zorial) is a specific inhibitor of the plant enzyme phytoene desaturase (PDS; Sandmann et al., 1989). To test the effect of norflurazon, its commercial formula Zorial was injected into mature green fruits of the *yellow flesh* mutant *r*^2997^. After ripening, diffused red sectors appeared in the treated fruit due to the accumulation of carotenoids (Figure 4A). A similar experiment was done in *ex-planta* fruit tissues. Disks of pericarp taken from mature green fruits of *yellow flesh r*^2997^ and the wild-type variety Rutgers were incubated *in vitro* and treated with Zorial. The Rutgers fruit disks appeared yellow to orange, as opposed to red in the untreated disks. The colors of the mutants’ disks were not changed compared to untreated control. However, carotenoid composition in the disks indicated that PDS inhibition induced phytoene synthesis in both *yellow flesh* fruit mutants *ex-planta* (Figure 4B).

**Figure 5:**
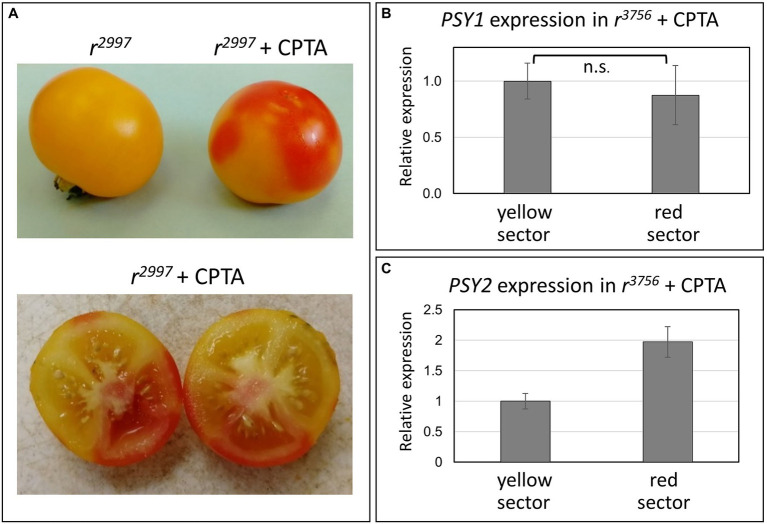
Effects of CPTA on pigment accumulation in tomato fruit. **(A)** Fruits of the *yellow flesh r*^2997^ mutant before and after CPTA treatment. **(B)** Expression of *PSY1* in the yellow and red fruit sectors. **(C)** Expression of *PSY2* in the yellow and red fruit sectors. Different tested parameters were compared using the “Compare all pairs” (Tukey–Kramer), *p* < 0.05. n.s. no significance.

The effect of inhibition of carotenoid biosynthesis downstream to lycopene was investigated in *yellow flesh* mutants treated with the lycopene β-cyclase inhibitor 2-(4-Chlorophenylthio)-triethylamine hydrochloride (CPTA; Sandmann et al., 1989; Cunningham et al., 1993). CPTA was injected to mature green fruits of mutants *r*^2997^, *r*^3756,^ and IL3-2 (*r^sp^*). In all mutants, the lycopene cyclase inhibition altered the fruit color in distinct sectors, which appeared red due to lycopene synthesis (Figure 5A, Table 2). However, the carotenoid composition varied among the different *yellow flesh* mutants. While in *r*^2997^, CPTA induced the accumulation of *cis*-carotenes, which led to red color appearance, in *r*^3756^ and IL3-2 (*r^sp^*) it caused the accumulation of mainly lycopene (Table 2). The expression of *PSY1* did not significantly change in the CPTA-treated sectors compared to the non-treated sectors. However, the expression level of *PSY2* was doubled (Figures 5B,C).

**Table 2:** Carotenoid composition in fruit sectors.

	Lutein	Lycopene	Prolycopene	Neurosporene	ζ-Carotene	β-Carotene	Phytoene + Phytofluene	Total
CPTA/*r^sp^* (IL3-2) yellow sector	1	0.2		0.1		0.9		2.2
CPTA/*r^sp^* (IL3-2) red sector	1.3 ± 0.2	68.6 ± 16.9					3.7 ± 1.3	73.6 ± 18.4
CPTA/*r*^3756^ yellow sector	0.7 ± 0.1			0.4 ± 0.1		0.3		1.4 ± 0.2
CPTA/*r*^3756^ red sector	0.6 ± 0.2	21.6 ± 2.4						22.2 ± 2.5
CPTA/*r*^2997^ yellow sector	1.6 ± 0.3	1.1 ± 0.7			2.5 ± 1	0.8 ± 0.3	8.8 ± 3	14.8 ± 5.1
CPTA/*r*^2997^ red sector	0.4 ± 0.1	0.8 ± 0.2	3.2 ± 0.4	2.6 ± 0.7	13.9 ± 1.4	1.2 ± 0.2	21.8 ± 2.6	46.4 ± 4.9

*Yellow and red fruit sectors following CPTA treatment of the yellow flesh alleles IL3-2, *r*^3756^ and *r*^2997^ were analyzed (μg g^−1^ FW)*.

**Figure 6:**
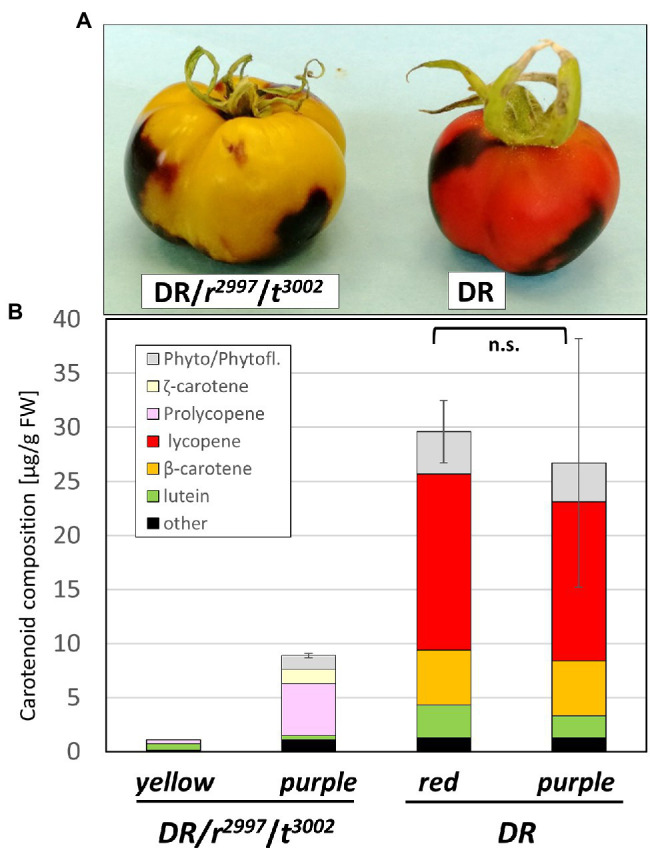
Transient silencing of *PSY2* in fruits of the double mutant *r*^2997^/*t*^3002^. TRV-based silencing of *PSY2* was done in the Moneymaker line expressing the *Delila Rosea* (DR) and in a triple mutant DR, *yellow flesh* and *tangerine* (DR/*r*^2997^/*t*^3002^). **(A)** Phenotype of fruit sector after co-silencing by VIGS of the *Del/Ros1* (*DR*) and the *PSY2* genes. **(B)** Carotenoid composition (μg g^−1^ FW) in different fruit sectors. The sectors were dissected from the silenced (yellow) and non-silenced (purple) of the quadruple mutant *DR*/*r*^2997^/*t*^3002^, and the silenced (red) and non-silenced (purple) of the *DR* plants as a control (right). The data were compared using the “Compare all pairs” (Tukey–Kramer), *p* < 0.05. n.s., no significance.

**Figure 7:**
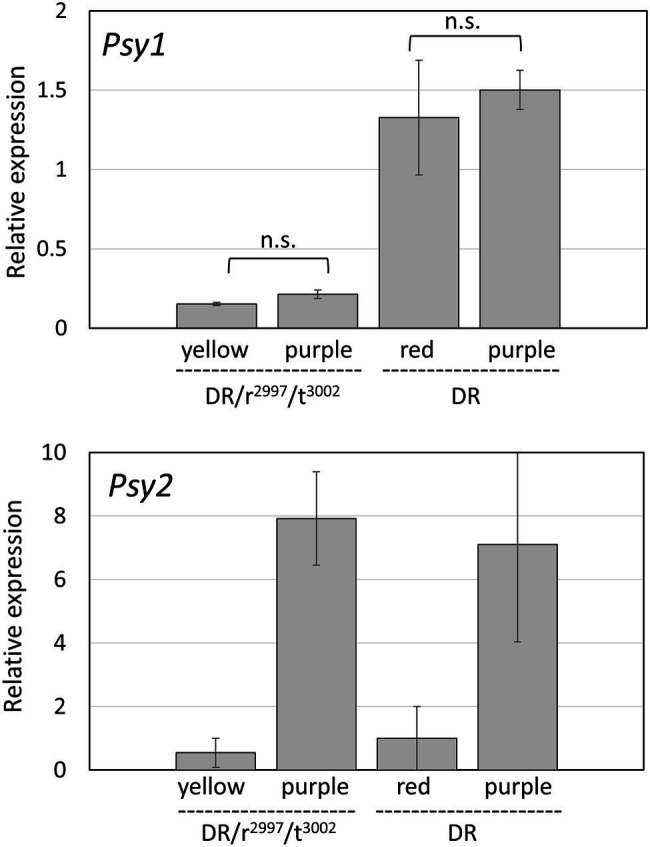
Transcript levels of *PSY1* and *PSY2* in silenced and non-silenced sectors dissected from VIGS-treated fruits of the *DR*/*r*^2997^/*t*^3002^ and *DR* (control) plants. The data were compared using the “Compare all pairs” (Tukey–Kramer), *p* < 0.05. n.s. no significance.

To investigate the potential involvement of PSY2 in the restoration of phytoene production in the double mutants of *tangerine* and *yellow flesh*, expression of the gene *PSY2* was inhibited by a transient VIGS. To this end, we created a tomato line that combined the mutations *yellow flesh r*^2997^ and *tangerine t*^3002^ with the transgenic line overexpressing the transcription factors Delila (*Del*) and Rosea1 (*Ros1*; DR), as visual reporters for silencing in tomato fruit (Butelli et al., 2008; Orzaez et al., 2009). The quadruple mutant line was named *DR*/*r*^2997^/*t*^3002^. A TRV-based silencing vector was constructed to silence both *PSY2* and the transgenes *Del* and *Ros1*. The Si-RNA sequence was designed to specifically silence *PSY2*, but not *PSY1* or *PSY3* (Materials and Methods). The silencing TRV vectors were injected to green fruits of the lines *DR*/*r*^2997^/*t*^3002^ and *DR* as a control. The silenced sectors were identified in ripe fruit by eliminating anthocyanins. In the *DR*/*r*^2997^/*t*^3002^ fruit they appeared yellow and in *DR* they were red (Figure 6A). Carotenoid analysis indicated that non-silenced tissues of *DR*/*r*^2997^/*t*^3002^ accumulated *cis*-carotenoids typical to the *r*^2997^/*t*^3002^ double mutant, whereas carotenoid synthesis in the *PSY2*-silenced sectors was nearly abolished (Figure 6B; Supplementary Table S2). Similar silencing of *PSY2* in the Del/Ros1 Moneymaker line did not change the carotenoid composition (Figure 6; Supplementary Table S2). Quantification of mRNA confirmed specific and efficient silencing of *PSY2* in the silenced sectors, while the expression of *PSY1* was unchanged (Figure 7). The elimination of carotenoid synthesis by *PSY2* silencing in the *DR*/*r*^2997^/*t*^3002^ line confirms that carotenoid biosynthesis recovery in the double mutant *r*^2997^/*t*^3002^ depends on PSY2 activity.

## Discussion

### The Origin and Molecular Basis of the *Yellow-flesh*, *r*^2997^

Several *yellow flesh* mutants in the locus *r* have been identified in tomatoes (Tomato Genetics Resource Cente).^2^ The allele *r*^2997^ (LA2997), which is described as a spontaneous mutation in the variety Rutgers, is considered the oldest allele (Chetelat, 2002) that was originated from the yellow variety brought to Europe in the 16th Century (McCue, 1952; Bergougnoux, 2014) and genetically characterized in the early 20th century (Price and Drinkard, 1909; Gilbert, 1912; Jenkins, 1948). We have confirmed that the mutation in *r*^2997^ is caused by an insertion of a *copia*-type retrotransposon *Rider* in the coding region of *PSY1*. The sequence of the insertion in exon #4 (Supplementary Figure S2) indicates a full-length *Rider* retrotransposon (Jiang et al., 2012). Previously, we missed identifying the mutation in *r*^2997^ while using genomic DNA sequencing based on PCR amplification (Kachanovsky et al., 2012). The existence of a complete retrotransposon bordered by identical long terminal repeats (LTR) often forms a stable stem-loop secondary structure. Most Taq-polymerases used in PCR amplifications skip stem-loop structures due to the replication slippage mechanism observed in the presence of direct repeats in the DNA (Viguera et al., 2001). Unlike previous analyses, here we used an engineered Taq polymerase, KAPA2G Fast, with higher processivity and speed under conditions that enabled the amplification of the full-length retrotransposon. LTR retrotransposons comprise more than 60% of the tomato genome. Most of them exist for millions of years in the exact chromosomal location (Tam et al., 2007; Cheng et al., 2009; Du et al., 2010; Jiang et al., 2012). Therefore, it is reasonable to assume that the retrotransposon mutation in *r*^2997^ is ancient and that it has prevailed throughout the tomato’s history since it was brought to Europe from America. The sequence of the retrotransposon LTR was previously detected in the GTOM5 mRNA from a *yellow flesh* mutation in the variety Ailsa Craig (NCBI Reference Sequence: X67143.1; Fray and Grierson, 1993a). Fray and Grierson (1993a) have predicted that this sequence belonged to a transposable element based on Southern blot hybridizations. According to the sequence of *PSY1* in *yellow flesh r*^2997^ (Supplementary Figure S2), it is evident that this is the same *r* allele described before in the Ailsa Craig variety (Fray and Grierson, 1993a). The phenotype of *yellow flesh r*^2997^ has been ascribed to the lack of *PSY1* transcription (Kachanovsky et al., 2012). This notion was supported by the fact that functional *PSY1* genes introgressed to *S. lycopersicum* from green-fruited wild tomato species exhibit *yellow flesh* phenotypes, as demonstrated by the allele *r^sp^* (Table 1). The existence of a 4.8 kb *Rider* retrotransposon in the first coding exon of *PSY1* explains the drastic reduction of mRNA measured by RT-PCR analysis based on the amplification of cDNA sequences downstream of exon #4.

### Reactivation of PSY2 in Ripening Tomato Fruit

The epistasis of the mutation *tangerine* over *yellow flesh r*^2997^ was attributed to increased transcription of *PSY1* in the double mutant *r*^2997^/*t*^3002^ (Kachanovsky et al., 2012). Loss-of-function mutations in the gene *CRTISO* were found to increase *PSY1* expression and enhance total carotenoids in fruits of *tangerine* tomato (Isaacson et al., 2002; Isaacson, 2005) and the *yofi* in melon (*Cucumis melo*; Galpaz et al., 2013). In both cases, the accumulation of *cis*-carotene intermediates could play a role in regulating gene expression (reviewed in: Cazzonelli et al., 2020; Escobar-Tovar et al., 2020). Measuring mRNA of *PSY1* in fruits of *r*^2997^/*t*^3406^ by RT-PCR amplification of exon #6 sequence showed a slight elevation compared to *yellow flesh r*^2997^ (Figure 3). However, this small increase in *Psy1* transcript cannot explain the restoration of phytoene synthase activity in the double mutant. This result differs from previous measurements in strain *r*^2997^/*t*^3002^ (Kachanovsky et al., 2012). The explanation for the discrepancy can be related to the different genetic backgrounds of *t*^3002^ (Rutgers) and *t*^3406^ (M82). Moreover, the *Rider* retrotransposon in exon #4 is inserted after codon 107 of the *PSY1* coding sequence. Consequently, the primary transcript of *PSY1* in *r*^2997^ consists of exons 1–3 and part of exon #4 fused to the LTR sequence of the retrotransposon (Figure 2). It thus potentially encodes a chimeric non-functional polypeptide of 178 amino acids. Other rare transcript variants of *PSY1* in *r*^2997^ skip exon #4, which encompasses the retrotransposon (Figure 2). However, a truncated PSY1 translated from exons 5–9 was inactive in the *E. coli* complementation assay (Supplementary Figure S4). These results indicate that the tomato mutant *r*^2997^ lacks a functional PSY1 enzyme and, therefore, the gene expression level is irrelevant. PSY1-specific antibodies are not available, and consequently, it was not possible to obtain quantitative data on the aberrant PSY1 protein level. The phenomenon of *tangerine* epistasis has also been observed with allele *r*^3756^ that carries a loss-of-function mutation in *PSY1* (Table 1). This result challenges previous conclusions derived from the double mutant *r*^3756^/*t*^3002^ on the epistasis of *tangerine* over *yellow flesh* (Kachanovsky et al., 2012).

**Figure 8:**
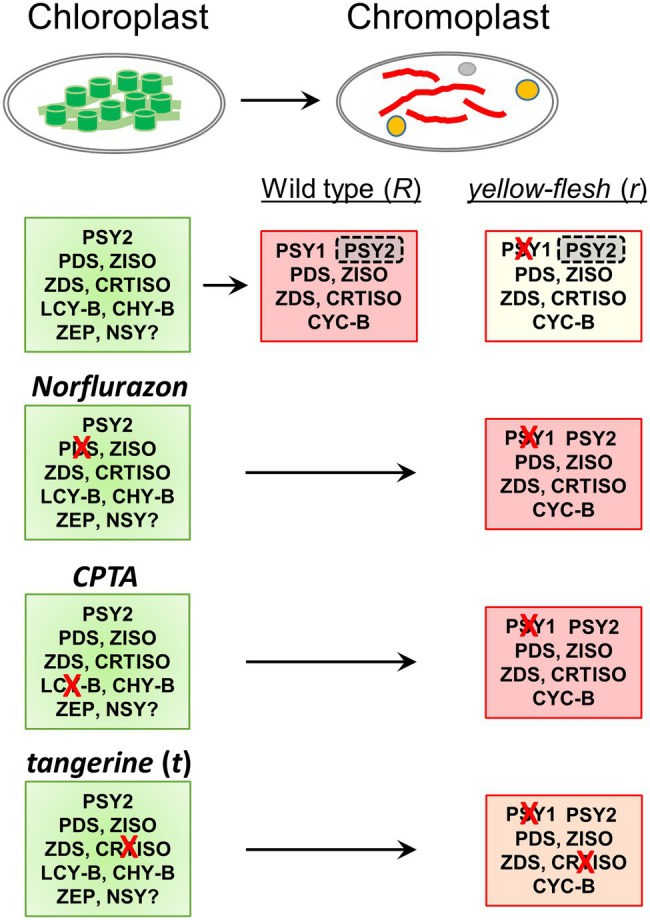
Summary of the experimental scheme and results. Carotenoid biosynthesis enzymes in tomato chloroplasts and fruit chromoplasts are depicted in the relevant boxes (see Figure 1 for enzyme abbreviations). Phytoene is synthesized in the chloroplasts by PSY2 and in chromoplasts by PSY1. PSY1 is exclusively expressed in fruits and flowers while PSY2 is found in both chloroplasts and fruit chromoplasts. However, PSY2 is inactive in chromoplasts (shaded) and, therefore, carotenoid synthesis is blocked in the fruits of the mutant yellow flesh. Injection of norflurazon or CPTA into mature green fruit inhibits the enzymes PDS and LCY-B, respectively. Consequently, PSY2 remains active after the transition of chloroplasts to chromoplasts during fruit ripening and sustains carotenoid biosynthesis in the mutant *yellow flesh*. The mutation *tangerine* inhibits the enzyme CRTISO and causes the accumulation of tetra-*cis*-lycopene (“prolycopene”). This perturbation activates PSY2 in the double mutant *tangerine/yellow flesh*.

The results of the experiments described here (summarized in Figure 8) support an alternative option for recovering phytoene synthesis in the *yellow flesh*/*tangerine* double mutants by activating the leaf-specific phytoene synthase, PSY2. *PSY1* is mainly expressed in the fruit, where its transcripts increase dramatically at the “breaker” ripening stage from practically undetectable levels at the mature green fruit. By contrast, *PSY2* transcript level is quite similar in green and ripening fruit (Fraser et al., 1999, 2007; Fernandez-Pozo et al., 2017; Shinozaki et al., 2018; Tomato Expression Atlas).^3^ Moreover, in *yellow flesh* fruit, the PSY2 protein has been detected immunologically, and its enzymatic activity of phytoene synthesis has been demonstrated *in vitro* (Fraser et al., 1999). Nevertheless, PSY2 is not active in the fruits when *PSY1* is impaired in *yellow flesh* mutants (Table 1; Fray and Grierson, 1993a; Fraser et al., 1999; Kachanovsky et al., 2012; Fantini et al., 2013; Chen et al., 2019; Zhao et al., 2020). The minuscule amount of carotenoids, mainly lutein and β-carotene, found in the fruits of *yellow flesh*, *tangerine* and *zeta* mutants, as well as in the norflurazon-treated wild-type fruits, are more likely residues from the chloroplasts prior to their transition to chromoplasts rather than a low basal activity of PSY2. An upregulation of *PSY2* transcription in the *yellow flesh*/*tangerine* double mutants was ruled out (Figure 3). The unequivocal evidence that PSY2 sustains phytoene synthesis in *r*^2997^*/t*^3002^ fruits is the outcome of *PSY2* silencing in these fruits. As seen in Figure 6, the lack of PSY2 abolished carotenoid biosynthesis in the double mutant. Why then PSY2 is not active in *yellow flesh* fruits, and what activates it in the genetic background of *tangerine*? It has been hypothesized that PSY2, which usually operates in chloroplasts, is not active in fruits due to failure to interact with other enzymes in the carotenogenic pathway in chromoplasts (Fraser et al., 1999). In such a case, *tangerine* could alter the carotenoid metabolon in chromoplasts in a way that enables PSY2 to function. One possibility is the localization of the enzyme in the plastids. The location of the biosynthesis enzymes, including PSY, is associated with the accumulation and sequestration of carotenoids (reviewed in: Li et al., 2016). A change in the PSY2 compartment within the plastids could enable its accessibility to the carotenoid metabolon in the chromoplasts. It was reported that PSY isozymes from various plants differ in their plastid sub-organellar localization, such as specific membranes or plastoglobuli (Shumskaya et al., 2012; Ampomah-Dwamena et al., 2015). The mutation *tangerine* eliminates carotenoids downstream to lycopene and causes an abnormal accumulation of *cis*-carotenes, mainly tetra-*cis*-lycopene (prolycopene). These changes in carotenoid constituent could modify the plastidial membrane organization, as seen in the Arabidopsis *ccr* mutants, which alter the membranous structures in etioplasts (Park et al., 2002). In this context, it must be emphasized that the effect of the *tangerine* mutation is already manifested in mature green fruit where significant amounts of *cis*-carotenoids accumulate in chloroplasts prior to their transition to chromoplasts (Isaacson et al., 2002).

Similar phenomena are likely to occur when carotenoid biosynthesis in fruits chromoplasts is interrupted by inhibitors. Fruits or pericarp tissues at the mature green stage treated with norflurazon accumulate upon ripening excessive amounts of phytoene (Figure 4; Filler-Hayut, 2012). The buildup of phytoene, which was begun in the chloroplasts, possibly affected the assembly of membranes during the transition to chromoplasts in a manner that enabled PSY2 functioning. The difference in the carotenoid composition following treatment with norflurazon between *ex-planta* pericarp and whole fruit of *yellow flesh* (Figure 4) can be attributed to the diffusion of the inhibitor in the fruits during ripening that dilutes it to a low concentration that no longer blocks PDS. Moreover, the borders of the fully inhibited fruit sectors are diffused and sometimes unclear.

Similar to norflurazon, the lycopene cyclase inhibitor CPTA also induced carotenoid biosynthesis in fruits of *yellow flesh r*^3756^ (Figure 5). Treatment with CPTA increased protein level of phytoene synthase in *Narcissus pseudonarcissus* flowers (Al-Babili et al., 1999) and caused transcriptional changes in *Citrus sinensis* (Lu et al., 2019). A slight increase of *PSY2* expression was measured in CPTA-treated fruit sectors while *PSY1* transcript levels were unchanged (Figure 5). However, since allele *r*^3756^ carries a null mutation in *PSY1*, the induction of lycopene synthesis must have occurred due to activation of the PSY2 enzyme. In green tissues, CPTA eliminates cyclized carotenoids and leads to an accumulation of lycopene and other intermediate *cis*-carotenes (Fedtke et al., 2001; La Rocca et al., 2007). These changes in *yellow flesh* fruits treated with CPTA at the green stages could alter sub-organellar structures in the chloroplasts that linger during their transition to chromoplasts and influence PSY2 activity in ripening fruits. Activation of phytoene synthesis in fruits of *yellow flesh* mutants as a result of lycopene cyclase inhibition by CPTA has been recently reported by Gupta et al. (2022). The authors discovered that CPTA did not influence the expression levels of carotenoid biosynthesis genes and that ectopic accumulation of lycopene in chloroplasts was associated with the transition from chloroplasts to chromoplasts and the activation of PSY2 (Gupta et al., 2022). A comparable but slightly different case exists in pepper (*Capsicum annuum*) where *PSY1* is the key enzyme responsible for fruit color and *PSY2* functions in leaves (Berry et al., 2019; Dyachenko et al., 2020; Wei et al., 2020). However, it was demonstrated that PSY2 activity contributes to synthesizing a basal level of carotenoids in the fruit when PSY1 is not functional (Jang et al., 2020).

It has been established in Arabidopsis, melon, and sweet potato that the DnaJ chaperon protein ORANGE (Or) stabilizes PSY and regulates its activity by direct interaction between the two proteins (Zhou et al., 2015). Differential interaction of Or with paralogous PSY enzymes was reported in saffron (Ahrazem et al., 2020). The activation of PSY2 prompted by changes in the carotenoid composition may be enabled by a unique interaction of Or with PSY2 that either stabilizes the enzyme or interferes with its import to chromoplasts (Yuan et al., 2021).

Interestingly, disruption of carotenoid biosynthesis at ζ-carotene in the mutant *zeta* (*z*^2083^) did not activate phytoene synthesis in *yellow flesh* fruits despite a significant accumulation of phytoene (Table 1; Kachanovsky et al., 2012). This phenomenon illustrates that only accumulation of a specific carotenoid intermediates are leading to activation of PSY2.

In conclusion, our results demonstrate that although PSY1 is the sole enzyme that produces phytoene in tomato fruit, under certain circumstances, PSY2 can be activated to sustain carotenoid biosynthesis. The results support the hypothesis on the existence of a carotenoid biosynthesis metabolon with distinct features in chloroplasts and chromoplasts. Furthermore, although transcriptional regulation is the primary mechanism determining carotenoid biosynthesis in tomato fruit, additional post-transcriptional mechanisms also play a role in this process. Additional future experiments that will determine the protein levels of PSY1 and PSY1 and their sub-organellar localization are required to elucidate the mechanism underlying the activation of PSY2 in fruit chromoplasts.

## Data Availability Statement

The original contributions presented in the study are included in the article/Supplementary Material, further inquiries can be directed to the corresponding author.

## Author Contributions

UK and JH conceptualize the research. UK, VM, and NB carried out the experiments. UK and JH wrote the manuscript. All authors contributed to the article and approved the submitted version.

## Funding

This research was supported by the Israel Science Foundation Grant No. 1930/18.

## Conflict of Interest

The authors declare that the research was conducted in the absence of any commercial or financial relationships that could be construed as a potential conflict of interest.

## Publisher’s Note

All claims expressed in this article are solely those of the authors and do not necessarily represent those of their affiliated organizations, or those of the publisher, the editors and the reviewers. Any product that may be evaluated in this article, or claim that may be made by its manufacturer, is not guaranteed or endorsed by the publisher.

## Footnotes

^1^ https://tea.solgenomics.net/expression_viewer/output

^2^ https://tgrc.ucdavis.edu/Data/Acc/GenDetail.aspx?Gene=r

^3^ https://tea.solgenomics.net/
