# Supplementary material for: Perturbations in the Carotenoid Biosynthesis Pathway in Tomato Fruit Reactivate the Leaf-Specific Phytoene Synthase 2
Source: Front Plant Sci. 2022 Feb 25;13:844748. doi: 10.3389/fpls.2022.844748 (PMC8914173; doi:10.3389/fpls.2022.844748)

Table S1. Primers of the markers used for the mapping of the mutation in *yellow-flesh r[2997]*

Marker name	Distance from PSY1 (bp)	Forward	Reverse	Type	Restriction enzyme
INDEL1	907865 upstream	5’-TCCAAAGAGGAAGGCATCAG-3’	5’-ATAGCAATAAGCCCGGTTTG-3’	length polymorphism (86 bp insertion in *S. pimpinellifolium*)	
INDEL839	210218 upstream	5’- GTGTCACGCAAATCGAAAAG-3’	5’-GCACGTGTGCACTTTCTCC-3’	length polymorphism (67 bp insertion in *S. pimpinellifolium*)	
INDEL850	96518 upstream	5’-AAGTTTTTACCACCTCAATTTTCC-3’	5’-TCAACGAACAATTAAAAAGAAGAAG-3’	length polymorphism (33 bp insertion in *S. pimpinellifolium*)	
INDEL8530	81339 upstream	5’-CGTTGTGCATATGACGGTTC-3’	5’-AGTCCAAGTCCATCAAAATCC-3’	length polymorphism (38 bp insertion in *S. pimpinellifolium*)	
INDEL856	44133 upstream	5’-CGTGGGATAAATCCCTAAATTG-3’	5’-AAAGCAAAGGGCACGAAAG-3’	length polymorphism (25 bp insertion in *S. pimpinellifolium*)	
858	31238 upstream	5’-CGATTGGAGCACTGTGGAC-3’	5’-TGAAGGGATGGCTTGTTTTG-3’	CAPS	SacI
8587	19208 upstream	5’-AAAGCCTGTTGAGAATGTTGTG-3’	5’-GGCGGTGCATATATAATCCTTAAC-3’	CAPS	AccI
8592	13440 upstream	5’-ATGGCGGTGGACATTGTAAC-3’	5’-TGAATTCTCCCAGTAAAGCATC-3’	CAPS	Hpy188I
ex4	11867 upstream	5’-GTTGATGGAGAATTTACAGTGTGC-3’	5’-TGTGAATTCGAGCAACATAGG-3’	CAPS	AluI
8597	8437 upstream	5’-GACAGTTGATCGTCGTTTCG-3’	5’-AAATTGCCATACTTCAGCTAGG-3’	CAPS	AseI
PSY1-5’	462 upstream	5’-CGACGAGGAGTAAGGTTTGC-3’	5’-GGTCCCTTTATATTTGGCACTAGG-3’	CAPS	AseI
8610	4688 downstream	5’-TCAATACAACAACATTTCTTCTTCC-3’	5’-TGGCTTGGATTGCTATTATTCTG-3’	CAPS	MboI
PSY1-3’	5834 downstream	5’-ACACACACCTGGGCATCAAGG-3’	5’-TTTCCCACCTAGTTTTTGGTC-3’	CAPS	MboI
INDEL882	215510 downstream	5’-GAGCTAAACGATGTCGTAATATACTG-3’	5’-AGCCATCACAAGCATAAAGTTC-3’	length polymorphism (29 bp insertion in *S. pimpinellifolium*)	
INDEL887	267150 downstream	5’-AATGATAATTAAACTCTTAGCCAAACA-3’	5’-TGAAATAAATCAACTTCGAACAAAA-3’	length polymorphism (30 bp insertion in *S. pimpinellifolium*)	
INDEL2	677990 downstream	5’- GTTCAAGGGTAAAAACGGTCT-3’	5’-CGAACTACGAATGCTTTTCCA-3’	length polymorphism (57 bp insertion in *S. pimpinellifolium*)	

Table S2. Carotenoid composition (µg.g^{-1} FW) in the *PSY2*-silenced and non-silenced fruit sectors of *DR/r^{2997}/t^{3002}* and *DR* (control) plants.

	Lutein	Lycopene	Prolycopene	Neurosporene	ζ-Carotene	β-Carotene	Phytoene + Phytofluene	Total
DR/r^{2997}/t^{3002} yellow sector	0.4±0.1		0.6±0.2	0.1±0.1			0.1	1.4±0.1
DR/r^{2997}/t^{3002} purple sector	0.4		4.8±0.3	0.8±0.1	1.3±0.2		1.3±0.1	8.9±0.1
DR red sector	3±0.6	16.3±2.9			0.2	5.1±0.7	3.9±0.3	29.4±2.8
DR purple sector	2±0.4	14.7±9.7			0.1±0.1	5.1±0.4	3.6±2.4	26.7±12.2

Table S3: The solvent gradient procedure used to separate carotenoids by HPLC at a constant flow of 1.6 ml/min.

Time (minutes)	Acetonitrile:H_2O (9:1)	Ethyl acetate
0-8	100%	0%
8-12	80%	20%
12-26	65%	35%
26-26.1	45%	55%
26.1-33	0	100%

Figure S1. Genetic mapping of the mutation *yellow-flesh r*^{2997} *PSY1*. A, Generating F3 recombinant lines from the crossing of *S. pimpinellifolium* and *yellow-flesh r*^{2997}. B, A scheme illustrating the fine mapping of the insertion in exon #4 of PSY1.

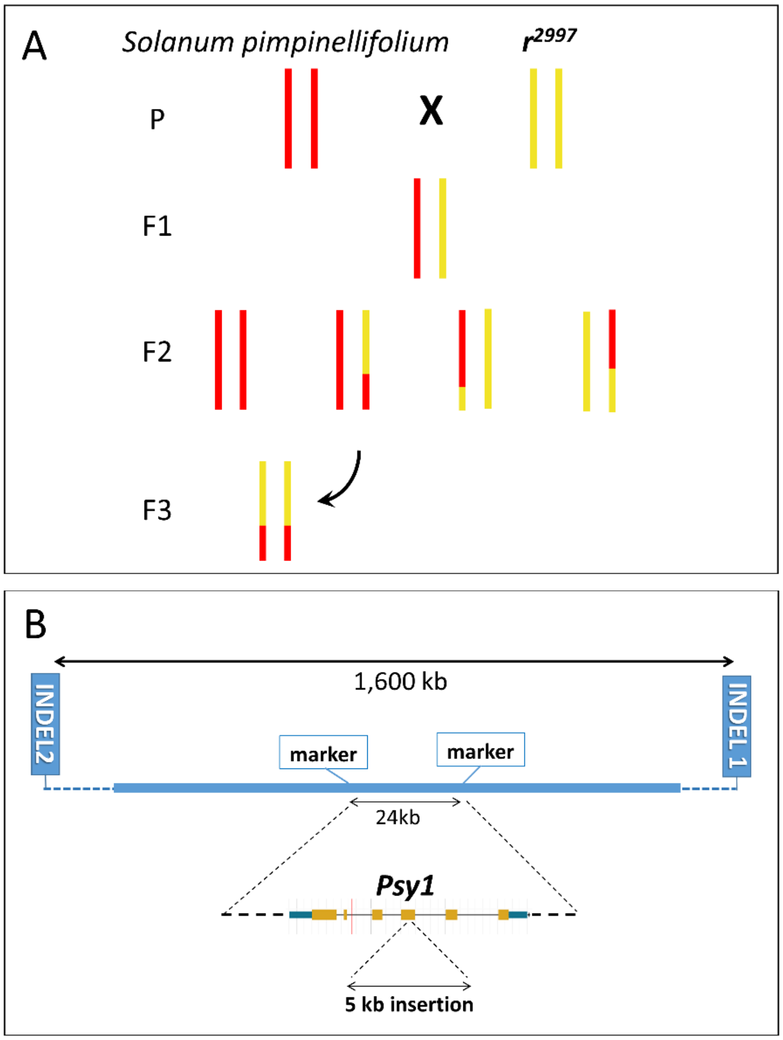

Figure S2: Genomic sequence of the *PSY1* gene from *yellow-flesh r*^{2997}. Nine exon sequences are highlighted in green. The ATG initiation codon in exon #4 is marked in red. The Rider retrotransposon sequence in exon #4 is in *Italics*. The 397 nucleotides LTR sequences are highlighted in gray.

aaatatagaatatccactaaggtggttacacgtgtacactcaaccaatgacggcacttcaaattcttgataacggta
tcttcccaccttcatatatttcaacattttattgttaaaataaaatcgcacgctctattttatctaaattttattta
tagaattatattaagtatgttattattaattttacgtataaatatatttcatattaaaatcatataagttgacatc
actaagttcgttggatttgcatgtagacccaattattcagatactcaacatgactcatttaatattggattattgaa
atatttgtaatacattaagaatataattgttaactattttaatttttaattatacgtaaatatcaaacaaaaatatt
aagtatgatcgctatattagatgataactataaggagcctacacaattaacactatttaactctattctttgcattt
ataaaaagttactttagtcttaggttcacaatgtcaaaatctaaacaactaaaaacgacgaggagtaaggtttgcaa
cgacgataacaaggattaggcaacaattagagttgtgaattgtgagtattaactatacttttactatattaggcaga
atttttgcactcaatgagtaacttgatttatttatttttatttcgccctaaattattggacaagtcatatatttgt
tttgaaaacattcttttattggctaaatcgaaaattgaatcgttaaagatcaaaaatcaataacaaatatcttattg
gtttaacatatttaaaaataaaaaaccaataaatctaactaataatatttaatacgaaaacgaaatggactgacaca
cattcctaaattttggtcaaaatttttcataatttccctaaaatctaaaatattaaatatttgacggaaacaaaa
aattcacttttaataaattatttgaaggactaaaacagtggaagaatatatttaagaagctaatttgaacctagtgc
caaatataaagggaccattttgtcatttttcaacttgaaaatctacgtgtcttaatatacaccaaagaattaatat
ttactgaaaaaatgtaaaaatgaggatatggattctgaatcactcaattccaatcagcaaaaataaaataaaataaa
ataaaataaaatttaaaaaataataataaatgctataaaatgaccaaaatgtgtggagcaaaaagtgcagaaaaaac
c**aacaaattgcattctccattcttggaagtggccattcttgatttcttgaaacaaag**gtttgtttcccttcacttct
tgatatgtaaagttgcaatctttataactttctattgctttgctag**tgtttttgttatatacaggggtggagttag**
agggtaagttacgcatttagtcgtaactttagtcaaacttcgtaataatttagtaagttaaaatatattagaaattt
tcagaattcataaactttaaatttaaattttgacttcgctttgtgtgactatacaattacag**aaattcagagtggc**
cattgttgaaagagagggtggaatttgtgtaagttttgtttcctttcagttcttgatatataaagttgcaatcttta
acattctttgttcactttctataggtttgctaggttcggttaaattcagtagctttagtttaaaccctatgcggaat
agagaatgtgtaaactttaaacttcaaattttggctccgcatacgactagcgactatataataataggaattgagca
cttggcttttgtatatagcttctatgtgtaccaaaattagaaaatcaggcgattattataatcttgttgactaaata
tagaatgcatccattacccccaaaaagtgtgattccactgtcataggaggtttttttatttcattttatttgtgct
ttcaataatgtagagtagttttacaaagatcctttctttgtgacacatggtaggtaatattgctgattttgttgtag
ttttggggttataaagtttcaaattatttatactggagggtaggggtgggggttgtctataatgcaggttatggttt
tacgtgaactcaataattattgtagatactaagaaatccactcagtgttcttgcggtgtcttgcttttgatttcagc
atcacttgtagttgattgtgtttagattatcacattattctgtggctgtaactgtatccttgttagttgctttgttt
ctacactgttgttttccctcttttatacctattttgatatgttgtactcgaacgagggtcatcggggaacaacctct
ttacctccgtgaggtagagctatggtctgtgtccactctaccctccccagatccctcttgtaggatttcactatatt
gtaatattaacttgaggtcactataggagctcaaaaacttctaattttgaatcaatgtctggttatacttttttttgt
cataactgtatctcaaatgtggtgtttggtttatctcattttgcag**aagtcaagaaacaggttactcctgtttgagt**
gaggaaaagttggtttgcctgtctgtggtctttttataatctttttctacagaagagaaagtgggtaattttgtttg
agagtggaaatattctctagtgggaatctactaggagtaatttattttctataaactaagtaaagtttggaaggtga
caaaaagaaagacaaaaatcttggaattgttttagacaaccaaggttttcttgctcagaATGtctgttgccttgtta
tgggttgtttctccttgtgacgtctcaaatgggacaagtttcatggaatcagtccgggagggaaaccgtttttttga
ttcatcgaggcataggaatttggtgtccaatgagagaatcaatagaggtggtggaaagcaaactaataatggacgga
aattttctgtacggtctgctattttggctactccatctggagaacggacgatgacatcggaacagatggtctatgat
gtggttttgaggcaggcagccttggtgaagaggcaactgagatctaccaatgagttagaagtgaagccggat***gttga***
atgccttgaatcggacccgctacaaacagaaaggacgggggtctcgctgcccggtcagcgagtcgggggtccaggg
gggcgacgcgccccctggcctgggggtccgggggggcggagacgcccccggccgacggtatacaatgttgttgtat
tgggcccttaattttctgttgattctgtatgttgggcccaagcctgttagggcgtagcttagcactatatatagacg
ctatgggaaaccctattctgtaattctgtttttgcctctccataataaaactgctccctctcttcccgtggacgtag
ccaatttgttggtgaaccacgtaaatctgttgtcttatttttcgcgtttatattttctcgtattatctcaaattccg
cacaacaaattggtatcagagcctctcggttaatcggtgttcttggagaattcgagatgtctgctttgaacgtgaaa
atcgacaaattcacagggaggaacagtttcagtttatggcagatcaagatgcgggccttgttgaaacagcaaggctt
ctgggcgccgttgtcgaaagacaagaacgccgtcgttactcctgagatggcgattctggaggaaaggcgcactcga
cgatcatgctgtgtctcgcggatgacgtcatcacggaggtctcggatgaagagactgctgctggtctgtggttgaag

ctggagagtttgtacatgacaaaatctctaaccaacaagctgcttctgaaacaacgtctattcggtttacgaatggc
tgaaggtacacaactcagggaacacttagagcaattgaatactttgttattagaattgcgtaatatcgatgtgaaga
tcgaggatgaagatgctgccctgattctgttagtatctctcccaatgtcgtttgagaattttgttcaatcgttcatt
gttgggaaagatactgtgtcactggaagaagtcagatcagcccttcatagcagggaattacggcataaggctaacgg
cacaagtacggacatacagccttccggtctgttcaccagtagcagaaagggaaggaaaaacggcggaaagaaaaata
agccgatgtcgaagggtgcaaagccggatgatgtttgtaattactgcaaggagaagggacattggaaatttgattgt
ccgaagaagaagaagcaatcggaaaaacaatcagtgtctgctgctgttgctgaagaagacaccaattctgaagaaga
tattgccctagttgcggatgagcacactcatcattcagatgtgtgggttcttgattctggggcatcctatcacatct
gtcctaggagagagtggttcacgacttatgagcaggtagacggaggcagcatctcgatggccaacagttctgtctgc
aaggtggttgggacaggctcgatcaagataaggacacatgacggtagcttctgcacattgaacgaggtcaggcacgt
tccattgatgacgaaaaatctgatatctctcagtcttttggacagcaagggattcagctggtcgggaaaagatggag
tcttgcgggtctggaagggttcaaatttgattctgaaaggtgtcatgcgtggtactttgtattttctacaaggttcc
acggttacaggttcagcccatgttgcatcgtcagaatttcaccagaaggatatgactaagttatggcacataagact
tggtcatatgggtgaaagagggatgcaaattctgtcaaaggaggatttacttgctggtcataaggttaagagcctag
agttttgtgaacattgtgtttttggaaaactacatcgcaacaagtttccaaaggccattcatagaacaaaaggcaca
cttgattatatccattctgattgctggggtccatgccgtgttgagtctttgggaggctgcagatttttttgtgtccat
tattgatgactactcaaggatgacttgggtgtacatgatgaagcataaaagtgaagccttccagaagttcaaggagt
ggaaaattttgatggaaaatcaaacagggaagaagatcaagaggttgcgaactgataatgggctggaattctgttgg
tctgaatttgatcaattctgtaaggatgaagggattgctcgacatcgcacagtcagaaatacaccacagcagaacgg
tgtagctgagcggatgaatcaaacacttctggagagagcaaggtgcatgctctctaatgctgggctagatagaagat
tctgggcagaagcggttagtacagcttgctacttgattaaccgcggaccacatacaggtatacagtgcaagacacct
atggagatgtggtctggaaaagctgctgattattcaaatctgaaagcttttggttgtacggcttactatcacgtcag
tgaaggtaagttagaaccaagagctaaaaagggagtatttgtgggctacggagatggagtgaaaggtttcagaatct
ggtctccagcagaaaagagggttattatgagcaggaacgttgtctttgatgaaagtcctctgcttagaaccattgtg
aagcctacaactacgtcagaaactgggagtcttgacaaacaggtggagtttcaagtcattcagaacgagagcgattt
aaaggaacctgaagaggaggatcaagagccacagactgaaactgatattccagaatctatgccatcagatatccatc
agagtatagctcaagatcggccaaggagggttggagttcggccacctacgaggtatggttttgaggacatggtgggt
tatgcactgcaggttgctgaagaggtagatacatctgagccgtctacttacaaagaagccattttaagttctgattc
tgaaaaatggtttgccgctatgggagatgagatggagtccctacacaagaatcagacatgggatctggtcatacagc
cttcggggagaaagattattacttgcaaatgggttttcaagaagaaggaagggatatcaccagcagaaggagtcaag
tataaagccagggttgttgccagaggtttcaaccaaagagagggagtggactacaatgagatcttctcaccagtggt
cagacatacttccatccgagtgttactagcgatagttgcacatcagaatctggagcttgaacaacttgatgtgaaga
cagcgtttctacatggagagttggaggaagagatatacatgactcagccggatggtttccaagttccagggaaggaa
aatcacgtctgcaagttgaagaagtccttatatggacttaagcagtctccaaggcagtggtataaaaggtttgacag
ctatatggtgaagttgggctatactcggagctcatatgattgttgtgtctactacaataggctcaatgatgattcat
tcatctatctggtgctttatgtagatgatatgttgatagctgcaaagaagaagtatgacattcagaagctgaagggt
ttacttagtgctgagtttgagatgaaggatctgggagccgctcggaagattttagggatggagatcattagagacag
agagagaaggaaacttttcttgtcacagagaagctacattcagaaggtcttggcgaggtttggcatgtcttcatcta
agcccattgataccccccagtgctgccaatatccatctcactgccatgttcgctccacagtcagaagaagagaaggag
tatatgtcacgagtcccttatgccagtgccgtaggaagtttgatgtatgctatggtctgtacaaggccagatttagc
acatgcagtcagtgtagtgagcagattcatgggacaaccagggagagaacattggcaggctgtgaagagaattttcc
ggtaccttagaggtacatctgacgttggtctcatttatggaggtgatactcagtgcttggttactggctattctgat
tcagactatgctggagatgttgacacaagaagatcgatgactggctatgtgtttacccttggaggatctgtcgtcag
ttggaaggcaactttgcaacctacagtgactttgtctactacggaagcggagtacatggcccttgacagaggctgca
aaagaaggatttggttgaaagggctggttagtgatcttggtctgcatcatgatcaggctacggtgtattgtgacag
tttgagcgcaatttgtctagccaaggatcaagtccatcatgagagaaccaagcatattgacgtaaggtatcattttc
taagaagtgagaagagaatcaaggtgaagaaagtaggaactgctgataatcctgctgatatgttcacaaagccggtt
ccacagagcaagtttcaacactgtttggacttgctcaacatcagaagctgttaattgccctgcggggcaactctgag
gaagaggggggaggcttggcactatcatagtgcgtctgaagaatctgttcggagaattcaagtcaaggtggagatttg
ttgaatgccttgaatcggacccgctacaaacagaaaggacgggggtctcgctgcccggtcagcgagtcgggggggtcc
agggggggcgacgcgcccccctggcctgggggtccggggggggcggagacgcccccgggccgacggtatacaatgttgtt
gtattgggcccttaattttctgttgattctgtatgttgggcccaagcctgttagggcgtagcttagcactatatata
gacgctatgggaaaccctattctgtaattctgtttttgcctctccataataaaactgctccctctcttcccgtggac
gtagccaatttgttggtgaaccacgtaaatctgttgtcttattttttcgcgtttatattttctcgtattatctcaaat
tccgcacaaca**atacctattccggggaatttgggcttgttgagtgaagcatatgataggtgtggtgaagtatgtgca**
gagtatgcaaagacgtttaacttaggttagcttcttcaatctattcattcgtttaccaaatattatttggtaagcac
taattatgaatatatatatgttcatgttattgatgaagacaaaatttgatctttgtttgtttattcag**gaactatgc**

taatgactcccgagagaagaagggctatctgggcaatatatggtgaggtttctagccatttaataacagttacgcgc
acaaacacatatgattaatcggggacgagaaaaaaagaaatgaagtttgagttttgagggtcatatgtaataggtaa
atccgagcttgactagcttgagatgtttattgtcatatcatgctcaatactaaccaaaacactgaaaaagaacttga
ttatatttacatactaatattttcatttgcgttgctgttcacatttttacctatggaactggtttttgtgatttgtt
atacttcatattcgatgttaataaaatatatcattcctcccttttttctccacttcaagctttactgtagtgttgaaa
ggggaaactccttttaatgattgcatatataaacgaacttcttgagttgaatagtttctcattatgatctgtttaaa
cag**tatggtgcagaagaacagatgaacttgttgatggcccaaacgcatcatatattaccccggcagccttagatagg**
tgggaaaataggctagaagatgttttcaatgggcggccatttgacatgctcgatggtgctttgtccgatacagtttc
taactttccagttgatattcaggttagtctaccaattctatggtctttatatttgttcaatttgcgtttgatgtcac
ttttgctgagggcttttctaatagcttacttcagcctagcggaaatgtttgtagttgaatctctagttctgtctcct
atatctgtttctctcgtcctagatactacacatacttcatttctgttttaacattttattcgtcttttggtgttgtt
ttgtatgtgaatcatatatttggaacagaatcattattagttcacatgatttcatttgctttcttcaatagcgtaat
tgtctaaccttccaatatatgttgcag**ccattcagagatatgattgaaggaatgcgtatggacttgagaaaatcgag**
atacaaaaacttcgacgaactatacctttattgttattatgttgctggtacggttgggttgatgagtgttccaatta
tgggtatcgcccctgaatcaaaggcaacaacagagagcgtatataatgctgctttggctctggggatcgcaaatcaa
ttaactaacatactcagagatgttggagaagagtaagtacaaagctgtgttttacgcacataatttttttttgctaat
atttacatatcaaaatataggaaaatgagctcttcggttatccggtttatattttttttatgtcaacataatagtat
aaagtaattagtatcagtcgttctgggaataaaattgcagaactcaatttagccgtgttgtgaaatcctgcttgttt
tgagagcttaaagctcattagttagtcgttagagacgaagaaattcttcgttgtccatctttattccaccttaaagt
tgtgatattttcattattggtacatttggcaaaaacacctgaacaaatttatgacggatgccttttgaaagtcacta
tacctgtctagtcggcgtttatcacatttctttgacatattgaactttgaaacatgatatcagctctagacagtgac
gagccatgatcaatttctttcctttattctttctttggaagtgccgtatttaggcttccgttgttcttatatattgc
tttccctgcag**tgccagaagaggaagagtctacttgcctcaagatgaattagcacaggcaggtctatccgatgaaga**
tatatttgctggaagggtgaccgataaatggagaatctttatgaagaaacaaatacatagggcaagaaagttctttg
atgaggcagagaaaggcgtgacagaattgagctcagctagtagattccctgtaagcattcgtaaactctttagtttt
atgaaatgattctttttcgcgttattagatgaatatggttgcttgtgttgagtatttctaggtcgatgaagttgag
acaagggttttaagttttaacgacttttacggggtgccatgttatctgctacctaatcttaggtagttgaccggaa
gggctagaatttaacctcatgttcaccctaccaaccaagaaatgaacctcgcatagagctcgtagttatgaatatt
tgctttggcatgacattgtgcggatcatgaaatgtcttagattatatggaaaaatcattctattacatcgaatagat
acattagatctaagaagcacgccgtgttgtaaatgagaaattctatagctcagatctttagttttctctgaacgacc
tacaaaccaacggataaccttgtattgagcttgtcgttctcagtatttgcactaacattacgtcgtgtggatcctga
aatggcttggattgctattattctggatatggcaaaaccattttattagtactagatatcgaataactacatttgac
cctacaagtaccctgggttggagttacaatatcccatacctcgtatctttagtgttctcttatttatcacctttgtc
tactattctggcaaaataacctcactcgttactcggtgttttccag**gtatgggcatctttggtcttgtaccgcaaaa**
tactagatgagattgaagccaatgactacaacaacttcacaaagagagcatatgtgagcaaatcaaagaagttgatt
gcattacctattgcatatgcaaaatctcttgtgcctcctacaaaaactgcctctcttcaaagataaagcatgaaatg
aagatatatatatatatatatatagcaatatacattagaagaaaaaaaggaagaagaaatgttgttgtattgatata
aatgtatatcataaatattaggttgtagtaacattcaatataattatctcttgtagttgttgtatcttcactttatc
tcaactcctttgagagaactttccgtagttatctgctttgcacttggttactcagaattttactgtgggcatgataa
ttgatataccaaattcagttttgattctatcgaaaaatttgttattacatttttttgggggaaaggaattatctca
gatatgacgattttacatttcaattttctaaagttgttatccaaattcgattcaatccatacaccaatatccaacaa
gagagttaagagacacattggtctagccatctatacgcagattattgagtagtatttttttaacagtgcaaacataat
tcggtttttttgtcgattcggtttgtagaagggaaaaatcacaatattttatagttgaatttgtaatctaaattcga
gttgtatgatacaagttgcttcgaatcgatcttgatacatatttctacctatcttaatagtcttttcaccttttttaa
aactttatatcaacaaaattagacatataaattgtaacgcaacatactttattaagtattggtggtttgggccaaaa
caaacaagatcatttaaggtatgagcaactctatcttgtatatttatttacggattatgattttattttaagaaaac
ttaataaaaatattattcaattacttttttttatatatgtatacacacacctgggcatcaaggaggaacgtaacata
caatatttcaatgaaaatactggtcgaatattgtttcatattatcatattgtataaatatcaatagaagatataata
ttattcagatgtggtatcaaggataaagaagtagtgattcaatgaatagatcaaaaggacaacagtaaaacaaatag
tttgggccgtaggctcaatcaaaacaaataaattaagttaagttcaatttgaataaatactttacgtaataaatcta
atttatataagttttaaaaaataatctcataaaatatttcgtgattattattctt

Figure S3. Gene structure of *PSY1* in *S. lycopersicum* cv M82. Three transcript sizes of PSY1 were detected in the indicated proportions (arrows).

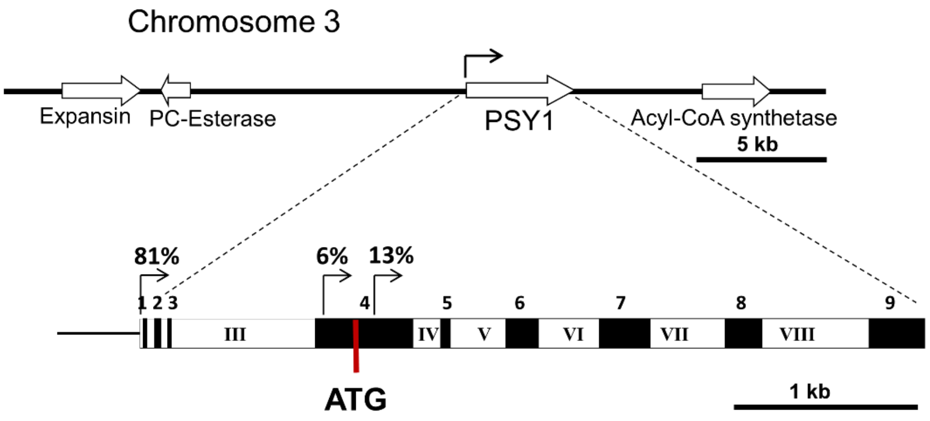

Figure S4: **Activity assay in *E. coli* of *PSY1* cDNA.** *E. coli* cells expressing CrtE (gernylgeranyl diphosphate synthase) and CrtI (phytoene desaturase) from *Pantoea agglomerans* were transfected with *PSY1* cDNA sequence comprising of exons 4-9 (left) or exons 1,3,5-9 (variant III in Fig. 2). The reddish color is caused by the accumulation of lycopene.

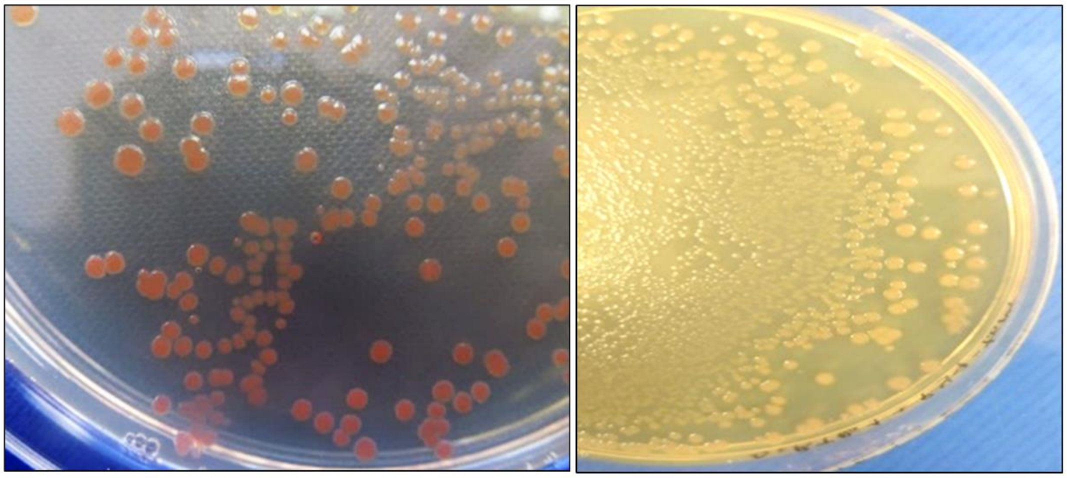

Figure S5: Ripe fruits of the *yellow-flesh* r^{3756} mutant and the double mutant *yellow-flesh* r^{3756} /tangerine t^{3002}.

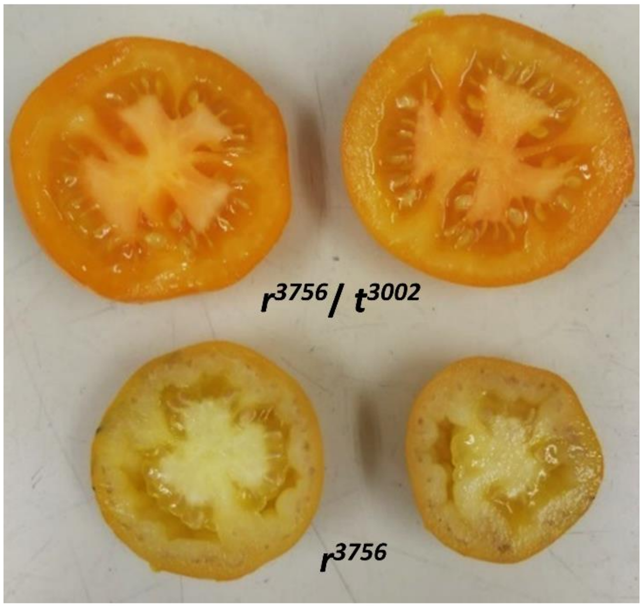

Supplement: Supplementary file 1 [file Data_Sheet_1.PDF]
